# Application of Radiomics in Predicting Treatment Response to Neoadjuvant Chemoradiotherapy in Locally Advanced Rectal Cancer: Strategies and Challenges

**DOI:** 10.1155/2022/1590620

**Published:** 2022-11-26

**Authors:** Rui Chen, Yan Fu, Xiaoping Yi, Qian Pei, Hongyan Zai, Bihong T. Chen

**Affiliations:** ^1^Department of Radiology, Xiangya Hospital, Central South University, Changsha 410008, Hunan, China; ^2^Department of Diagnostic Radiology, City of Hope National Medical Center, Duarte, CA, USA

## Abstract

Neoadjuvant chemoradiotherapy (nCRT) followed by total mesorectal excision is the standard treatment for locally advanced rectal cancer (LARC). A noninvasive preoperative prediction method should greatly assist in the evaluation of response to nCRT and for the development of a personalized strategy for patients with LARC. Assessment of nCRT relies on imaging and radiomics can extract valuable quantitative data from medical images. In this review, we examined the status of radiomic application for assessing response to nCRT in patients with LARC and indicated a potential direction for future research.

## 1. Introduction

Colorectal cancer is the third most common cancer in men and the second most common cancer in women, and it is expected to increase by 60% in 2030 [1], with rapid rising morbidity and mortality rates in many low- and middle-income countries [1]. About 30% of patients with colorectal cancer have rectal cancer [2]. Locally advanced rectal cancer (LARC) is defined as rectal cancer with clinical tumor stage 3-4 (cT3-cT4, tumor invading through the muscularis propria) or positive clinical nodal stage (cN+, malignant lymph nodes detected) [3]. Neoadjuvant chemoradiotherapy (nCRT) followed by total mesorectal excision (TME) is the standard treatment for patients with LARC [4], with the goal to eradicate micrometastatic diseases and to improve survival. For patients with LARC, the responses to nCRT vary widely, from a pathological complete response (pCR) to almost no response or tumor progression in a small group of patients [5, 6]. Approximately, 20% of patients will achieve pCR after surgery [5], which is defined as a complete response without residual tumors on the histological report after standard excision [7]. Currently, the only accurate way to confirm pCR is the pathological diagnosis after TME [8]. Thus, a feasible and accurate preoperative noninvasive prediction method will be helpful for assessing the efficacy of nCRT and for developing a personalized treatment plan [9]. Moreover, patients with favorable prediction results can be treated with an organ sparing therapy to ensure quality of life [10] and presurgical prediction could aid in the selection of the best therapies [11].

Magnetic resonance imaging (MRI) and computerized tomography (CT) are the most common imaging modalities for patients with LARC. However, the traditional imaging characteristics discernible with human eye such as tumor size, location, enhancing characteristics, etc., could not effectively predict the treatment response to nCRT. Advanced functional imaging methods such as diffusion-weighted imaging [12] have been shown to improve response assessment but could be costly and time-consuming. Therefore, a noninvasive approach for predicting the response to nCRT based on the initial pretreatment MR or CT images could potentially assist clinical management.

Radiomics has emerged as a promising tool for assessing imaging biomarkers to treatment response and it is defined as a method of extracting image features from medical images with high-throughput [13]. Radiomics involves data acquisition and preprocessing, tumor segmentation, feature extraction, and modeling. Compared to the traditional visual methods for qualitative imaging features that are discernible to human eye, radiomics uses novel computational techniques to mine the quantitative features contained in medical images. Radiomics provides convenient, repeatable, and objective information that can more effectively assist in clinical decision making.

Over the past decade, there has been an increasing number of radiomic studies on rectal cancer; approximately, 60% of the relevant radiomic literature on LARC focused on the prediction of treatment response and long-term prognosis after preoperative nCRT [11]. These studies addressed various aspects of rectal cancer, including pCR after nCRT, decrease in staging after nCRT [14], lateral lymph node metastasis [15, 16], and extravascular invasion [17]. Radiomic parameters, such as skewness, entropy, kurtosis, and evenness, have been used to assess and quantify intratumoral heterogeneity [18], which could potentially make up for the lack of spatial heterogeneity in the TNM staging system [19]. Previous radiomic literature on rectal cancer has identified various features and predictors for tumor response in divergent studies that differed in study design (multicenter versus single center, retrospective versus prospective), image segmentation method (manual versus automatic segmentation), imaging modality (CT, MRI, or PET), and predictive modeling (machine learning versus deep learning methods) [3, 9, 20–24]. Nevertheless, there is still no consensus among the researchers regarding the optimal application of radiomics to predict treatment response for LARC.

This review comprehensively assessed the body of literature on radiomics and its application in rectal cancer regarding treatment response to nCRT. Radiomic analysis such as data acquisition, tumor segmentation, feature extraction, feature selection, and predictive modeling was also reviewed. Through the literature review, we identified potential areas for future research. The studies included in this review are presented in Table 1 [3, 9, 12, 14–18, 20–76].

## 2. Radiomic Methods

**Figure 1:**
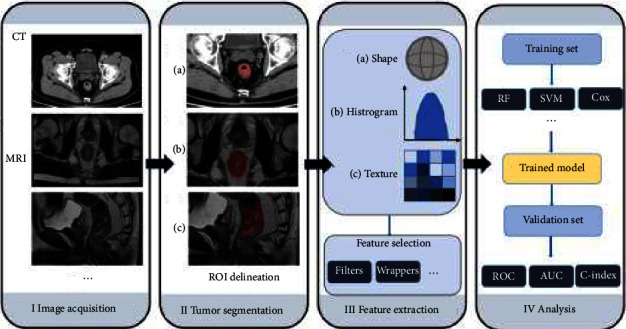
Representative pipeline of radiomic analysis for rectal cancer. CT: computer tomography, MRI: magnetic resonance imaging, ROI: region of interest, RF: random forest, SVM: support vector machine, Cox: cox regression, ROC: receiver operating characteristic curve, and AUC: area under curve.

**Figure 2:**
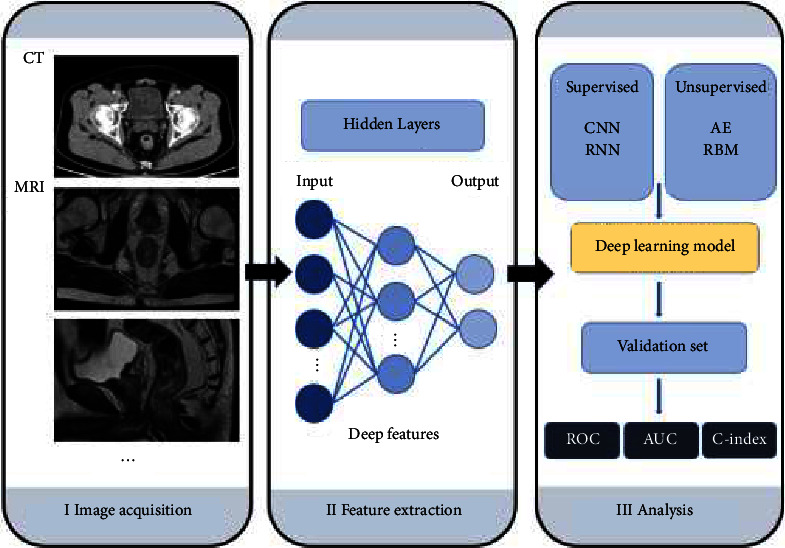
Representative pipeline of deep learning-based radiomic analysis for rectal cancer. CT: computer tomography, MRI: magnetic resonance imaging, CNN: convolutional neural networks, RNN: recurrent neural networks, AE: autoencoder, RBM: restricted Boltzmann machine, Cox: cox regression, ROC: receiver operating characteristic curve, and AUC: area under curve.

Radiomics can be divided into classic radiomics and deep learning-based radiomics depending on if a deep learning technique is used. Classic radiomics involves the following steps: image acquisition and preprocessing, tumor segmentation, feature extraction, feature selection, modeling, and validation (Figure 1) [24]. Deep learning-based radiomics performs learning procedures *via* convolutional operations such as the convolutional neural network (CNN) approach (Figure 2) [75].

### 2.1. Classic Radiomics

Data acquisition is the first step in classical radiomic methods. Data used in radiomic studies may be retrospective or prospective from a single-center, or a multicenter setting. The image modality can be CT, MRI, and PET. The specific choice of modality needs to be decided according to the research objective. The investigator should first identify the clinical problem to be addressed and should be aware that imaging protocols may not always be standardized with variability between institutions. In this regard, the recommendations of the Image Biomarker Standardization Initiative may help to reduce the variability of image preprocessing before analysis [76].

To achieve repeatability and generalization, several preprocessing steps after image acquisition are necessary, which typically include the following: intensity normalization, spatial smoothing, spatial resampling, noise reduction, and MR field nonuniformity correction [77].

Tumor segmentation is another basic step in radiomics, where researchers typically analyze the entire primary tumor and select the region of interest (ROI) corresponding to one slide of the image or a volume of interest (VOI) indicating the volume of a specific area. Image segmentation of the target ROI or VOI can be done manually, automatically, or semiautomatically. Manual segmentation is more accurate in some cases, but less repeatable. Automatic segmentation depends on the algorithm, which is efficient and helpful to eliminate subjective errors. However, there is a lack of accurate automatic segmentation algorithm so far, and its application is limited. Currently, several steps have been used to improve radiomic performance, including involvement of different medical professionals, adaptation of consistent methods in tumor segmentation, and standardization of imaging features [78].

Based on ROI or VOI, radiomic features are extracted from the images. These quantitative imaging features are important characteristics in radiomics because they bridge medical images and the clinical endpoint. These intrinsically valuable features can be extracted directly from the initial medical image, or by transformation or filtering. This process can be performed using different open-source tools such as PyRadiomics, TexRAD, and MaZda, and the main method is based on the study published by Aerts et al. [79]. Different types of quantitative features can be extracted from medical images; these features are mathematically defined differently and features are usually divided into the following subgroups. Shape features represent geometric relations and properties of the segmented ROI or VOI, such as the maximum diameter, maximum surface area, volume, compacity, or sphericity [80]. First-order statistical features or histogram-based features use the image intensity distribution represented by histograms that characterize the distribution of individual pixel or voxel-intensity values within the segmented ROI or VOI. Second-order statistical features or textural features quantify the intratumoral heterogeneity. Higher-order features are usually statistical features computed on matrices that consider relationships between three or more pixels. In addition, wavelet features and model-based features are also used in radiomic studies [53, 71].

In radiomic analysis, feature selection is a necessary step to obtain features closely related to target results. Hundreds or thousands of features are often extracted, a large proportion of features may not be useful for the task and unstable features should be excluded to preserve the most important features and prevent overfitting. Therefore, feature selection is a critical step in radiomics. The commonly used feature selection methods in radiomics are divided into three broad categories: filters, wrappers, and inserts [81]. Minimum absolute shrinkage, selection operator regression, minimum redundancy, and maximum correlation are commonly used algorithms for feature selection.

After feature selection, it is necessary to establish a prediction model, which usually includes biological, imaging, and clinical feature parameters. Machine learning provides several modeling methods. The most used methods in radiomics are linear and logistic regression, decision trees (e.g., random forests), support vector machines, neural networks, and Cox proportional risk models. Each modeling approach has its limitations. In logistic regression, the Bayesian networks and deep learning, feature independence, feature discretization and network configuration dependence should be considered, respectively. In building the model, researchers can use different software tools such as the R-language and the SPSS modeler [82].

The models can be validated internally and externally. In addition to validation of the model, quality assessment should be performed to ensure reproducibility of the study. A model may be used potentially for clinical decision making only after a standardized assessment of its performance has been completed.

### 2.2. Deep Learning-Based Radiomics

Deep learning is a deep neural network architecture based on broad spectrum algorithms that allow machine learning of highly complex mathematical models for data representation and for performing accurate data analysis. Manual and semiautomatic methods are time-consuming and difficult to implement in clinical practice with a high degree of intraobserver and interobserver variability [83]. In addition, feature selection using the filter mentioned above is also time-consuming and laborious. On the other hand, deep learning can help to solve some of the issues associated with classic radiomics. Deep learning methods often rely on information about outcomes to select their features. In contrary to the classic radiomics, deep learning-based radiomics skips the steps of image segmentation and feature extraction. Instead, it uses the entire non-segmented image to extract and select high-dimensional features through the automatic neural network and to identify the inherent information contained in the images without manual segmentation [84]. The following three types of deep learning models are commonly used for medical imaging: convolutional neural networks (CNNs), generative adversarial networks, and sparse autoencoders. Deep learning-based radiomics performs the learning process through the convolutional operation and the CNN structure [82]. Compared with traditional radiomics, convolution operation has a stronger feature extraction ability. In deep learning models, deep learning features are usually extracted from convolutional layers. By changing the convolution kernel and modifying the structure, the neural network structure can flexibly extract different task-related features, thus making the method more targeted. Each hidden layer module in the network transforms the representation at a level. For example, the first level may represent edges in an image oriented in a particular direction, the second may detect motifs in the observed edges, and the third could recognize objects from ensembles of motifs [85].

Similar to the classical machine learning methods, deep learning also has supervised, unsupervised, and semisupervised methods. Supervised deep learning methods include CNN and recurrent neural networks (RNN), which use their internal memory to process sequential inputs and take previous outputs as the input. Through learning, these methods could assess which data in the sequence is important and should be kept or discarded. Unsupervised learning algorithms include deep auto encoders (AE) and restricted Boltzmann machines (RBM) [75].

Deep learning-based radiomics also has its limitations. The main issue is the need for large datasets to train the model because feature selection depends on training data rather than hand-crafted radiomics. Another issue is the lack of interpretability. Artificial neural networks build complex computational functions that can be challenging to interpret as the generated features are not easily explained by tumor characteristics. A comparison between classical radiomic methods and deep learning-based radiomic methods is summarized in Table 2.

## 3. Radiomics for Predicting Response to nCRT in Patients with LARC

### 3.1. Image Acquisition

In this review, studies on patients with biopsy-proven non-mucinous LARC were included. Studies with poor image quality, incomplete tumor coverage on imaging, rectal perforation, or mucinous tumors were excluded.

Most radiomic studies on LARC used MRI and CT images and few used PET/CT images. MRI is commonly used for imaging rectal cancer because it has the advantages of no radiation and high soft tissue resolution, which can clearly identify the rectal wall [86]. In the MRI radiomic studies, T2-weighted imaging has been used as a morphological parameter. Other multiparametric MRI such as a combination of diffusion-weighted imaging (DWI), T2-weighted imaging, and dynamic contrast enhancement imaging have also been used. For staging, MRI provides tissue details about the tumor location, extension, and relationship to surrounding tissues to establish markers for subsequent treatment. In addition, it reveals prognostic information such as mesenteric fat involvement, vascular invasion, and distance to the anal sphincter complex [87].

CT imaging is capable of predicting lymph node metastasis [88], but a few studies have indicated otherwise [15–18, 29, 59, 60, 64, 71, 89]. The CT radiomic model built by Jiazhou Wang et al. improved the prediction ability of overall survival to 0.730 from 0.672 with only clinical characteristics in patients with LARC treated with nCRT [71]. Hamerla et al. showed that random forest classification added no value to radiological data obtained from non-contrast CT scans in patients with rectal cancer [64]. Vandendorpe et al. predicted the clinical response to nCRT using contrast-enhanced CT and texture analysis, reaching an area under the curve (AUC) of 0.70 [14].

Six studies focused on PET/CT-based radiomics to predict the treatment response [27, 31, 33, 48, 58, 70]. 18-F [FDG]-PET/CT was used in radiomic studies where texture analysis was performed. Giannini et al. reported that their logistic regression model could predict the complete response with an AUC of 0.84, with higher gray-level co-occurrence matrix (GLCM) contrast and lower GLCM homogeneity [27]. Shen et al. developed a random forest model based on 18-F [FDG]-PET/CT to predict pCR after chemoradiotherapy in rectal cancer [48]. Martin-Gonzalez et al. assessed tumor heterogeneity in 18-F-FDG PET [33, 90].

### 3.2. Tumor Segmentation, Feature Extraction, and Selection

In the literature presented in Table 1 [3, 9, 12, 14–18, 20–76], most researchers used manual segmentation, in which two experienced imagers were generally responsible for drawing tumor ROI. A few used automatic tumor segmentations. Jin et al. proposed a multitask deep learning approach to predict treatment response and tested a model in a multi-institutional cohort of patients with rectal cancer. The deep neural networks were performed on two different but related tasks simultaneously, namely, tumor segmentation and response prediction. The tumor segmentation of the proposed network was consistent with the expert description and the results were similar to the specialized deep neural networks trained with a single task. The AUC values from internal and external validation cohorts for predicting treatment response were 0.95 and 0.92, respectively [21]. Leng et al. developed endorectal co-registered photoacoustic microscopy (PAM) and ultrasonography system paired with a CNN to assess the rectal cancer treatment response, which enabled automatic ROI selection [22]. Pang et al. introduced a deep learning model for ROI characterization. A novel two-stage model, called two-stage rectal perception U-NET (TSRAU-NET), was proposed to replace manual assessment. Their results with AUC values of 0.829 and 0.815 from the internal and external validation sets validated the feasibility and stability of their method for pCR prediction [9].

Regarding tumor segmentation, it has been suggested that intraclass correlation coefficients (ICC) should be used to assess inter-reader and intrareader consistency [91]. In addition, given the possibility of subjective bias, segmentation results may be inconsistent, which may be mitigated by providing more training to imagers, or performing multiple segmentations [78].

Extracted radiomic features include features for intensity, shape and size, texture, and for wavelet and Gabor filters [28]. Zwanenburg et al. standardized 174 radiomic features to enable verification and calibration of different radiomic software [76]. Their dataset consisted of features commonly used to quantify morphologic characteristics, first-order statistical aspects, and spatial relationships between voxels (texture) in three-dimensional images of the regions of interest (ROI). The commonly used platform is py-Radiomics, a flexible open-source platform that extracts a large number of engineering features from medical images, which enables standardization of feature definition [37].

Delta-radiomics is another radiomic method that extracts features from a time series of images to reflect the time variation of radiomic features [92]. For instance, this method has shown an improvement over the radiomics that focus on a single time point for assessing overall survival in patients with recurrent glioblastoma [92]. A recent study showed that the T2-weighted imaging-based delta-radiomics improved the early response assessment in patients with soft tissue sarcomas [93]. A study by Davide et al. identified two delta-radiomics features including the change in minimum length of principal component analysis (△Least) and gray inhomogeneity calculated by a run matrix (△GLNU) as the promising predictors of clinical complete response to nCRT in patients with rectal cancer [53].

Most radiomics studies have used a filter for coarse radiomic feature selection [91]. Filter methods can be generally divided into two types as follows: univariate methods and multivariate methods. Univariate filters rank features using the Chi-square test or the Mann–Whitney *U* test. A multivariate filter consists of a collator and a subset selector. Another feature selection method commonly used in radiomics is the least absolute shrinkage and selection operator (LASSO) method; which is a linear regression contraction and selection method proposed by Tibshirani [94]. In the study by Yi et al., MaZda software was used for the first time to generate a total of 340 quantitative features, and the LASSO method was then used to select the most useful predictive features from the original dataset. Radiomic score (RAD-Score) was calculated for each patient, weighted by their respective coefficients as linear combination of selection features [95]. Several studies used a combination of methods for feature selection. For instance, a study by Pang et al. adopted two feature selection methods in their study, i.e., first calculating Harrell's Concor index (C-index) between the feature and the pCR status to evaluate the discriminating ability of each single feature and then using the LASSO method to further select the remaining features [9].

In almost all the studies included in this review, manual segmentation was performed, which was laborious and time-consuming. Various feature reduction methods were used to ensure the number of features being reasonable compared to the number of enrolled patients, thereby reducing overfitting or type I errors [11].

### 3.3. Modeling and Validation

Radiomics aims to construct predictive models for clinical outcomes. In machine learning, several algorithms can be used to generate predictive models. Validation is an integral part of a complete radiomic analysis. There is no doubt that independent external validated models are more reliable than internally validated models because the results of data obtained independently are generally more reliable. The receiver operating characteristic curve (ROC curve), sensitivity, and specificity of the model can be used to measure the performance of the radiomic model.

There are usually two datasets in radiomic analysis, i.e., the training dataset (for training the model) and the validation dataset (for evaluating the model performance). The validation sets can come from external or internal sources although few studies used external validation data due to unavailability. Most studies were retrospective and had a small sample size without external validation. Nevertheless, it should be stressed here that radiomic studies with independent external validation are more reliable than studies with only internal validation. Radiomic results from externally validated studies are generally more robust and more applicable to clinical practice.

### 3.4. Deep Learning-Based Radiomics

A few studies have used deep learning to predict treatment response in patients with rectal cancer [3, 9, 20–24]. Trebeski et al. constructed a deep learning model to segment rectal tumors by fusing diffusion-weighted imaging (DWI) and T2-weighted imaging (T2WI), and obtained dice similarity coefficient (DSC) values of 0.70 and 0.68 [96]. Zhu et al. proposed a deep learning model for automatic segmentation of rectal tumors on DWI images, constructing a 3D volume U-net to characterize the spatial features in all three directions, unlike the previous DWI and T2WI fusion model. This model was designed to perform segmentation using DWI data alone to avoid potential registration errors [97]. Leng et al. developed an imaging system consisting of an intrarectal registration photoacoustic (PA) microscope (PAM) that was paired with a convolutional neural network (CNN), which showed high diagnostic performance in assessing the treatment response with potential for optimizing posttreatment management [22]. However, a study by Khadidos et al. evaluated six traditional learning models and one deep learning model based on MRI texture analysis of patients with LARC, and found that their deep learning CNN model did not show any predictive potential [24].

It should be noted that deep learning requires more data than traditional machine learning. In addition, if data from more than one scanner were used, magnetic field or vendor signal variability should be taken into consideration. A study based on two 1.5 Tesla MR scanners found that 75% of functionality was unstable due to vendor and image acquisition variability [49]. If the images were scanned at different magnetic fields, the changes would be even greater. Since few radiomic studies of LARC used deep learning, the information regarding its validity and efficacy are limited and more studies should be performed to fully assess its potential for clinical applications.

## 4. Discussion and Future Perspective

In this review, we presented data to show radiomics as a promising noninvasive imaging-based method for predicting treatment response in patients with LARC. Future radiomic research should focus on independent validation of existing models while continuing to develop new models for novel research questions. The current knowledge on deep learning in LARC is limited and more research is needed to explore its potential for clinical applications. Incorporation of multimodal imaging data and other factors such as clinical features and surgery-related variables should enhance predictive model performance.

There are inherent issues with interpretability of radiomic models. A lack of understanding of how machine learning predictions are generated remains a barrier to its adoption in clinical practice. This situation also occurs in deep learning approach. These black-box-like networks are hard to understand and hard to correlate with clinical outcomes with no strong theoretical support [98]. The lack of interpretability of predictive models can undermine interest and trust in them [99]. More work needs to be done to familiarize the users of radiomic models and help them to understand the associated interpretations [100]. Multidisciplinary teams need to create visual displays to help clinicians better understand how machine learning works [101].

The high variability of data acquisition should also be addressed in radiomics. As imaging protocols and scanner parameters are different in various research centers, the radiomic results are often different, which will greatly affect the reproducibility of radiomic data. It is prudent to use multicenter studies and standardized imaging parameters [46]. MRI and CT are commonly used as imaging modalities of choice for patients with rectal cancer. Staal et al. evaluated quality of published literature using Quadas-2 and radiomics quality score (RQS). They concluded that the high-quality studies were predominantly MRI-based radiomic analysis of the rectum [11]. Although CT may not have the detailed tissue characterization as the MRI for assessing treatment response of LARC, CT is still more commonly used than MRI in clinical practice. Nevertheless, the combined imaging features of CT and MRI are desirable [37]. The multimodal radiomic model designed by Li et al. achieved an AUC of 0.925 in the training set and AUC of 0.93 in the validation set [89]. However, the multimodal strategy is time-consuming and costly with a potential issue for overfitting.

There were issues with the study design of radiomic studies. Most of existing radiomic studies are retrospective, sourcing from single-center data with the lack of independent external validation, which may limit the reliability and applicability of the results. Multicenter studies are conducive to reducing bias yet not achieved in most studies.

There was a lack of clinical features in predictive modeling in the studies included in this review, suggesting a need to take clinical features into consideration for future studies. For instance, preoperative factors [57], surgical approach, and postoperative treatment all have important effects on the prognosis of patients with LARC [102], which should be included in modeling. In addition, studies incorporating clinical features have a higher predictive value. Pizzi et al. presented a novel machine learning model combining clinical and MRI-based radiomic features, demonstrating that the combination of clinical and radiomic features contributed to improved performance of the prognostic models [37]. Their result was generally in line with that of a study by Staal et al. [11]. This review was limited due to the lack of surgery-related literature for prediction models of surgical planning, preoperative and postoperative complications. Future radiomic studies and predictive modeling should incorporate relevant surgical information to improve model performance for predicting LARC prognosis.

## 5. Conclusion

In summary, this review examines the status of radiomic application in predicting treatment response to nCRT for patients with LARC. The limitations of existing radiomic studies have pointed out the need for large-scale prospective multicenter approach to avoid the potential pitfalls of small sample size, single-center data, imaging variability, and overfitting issues. In addition, there is a need to incorporate clinical factors in predictive modeling to improve the model performance and clinical relevance. More work needs to be done to render the radiomic data more interpretable and explainable to enhance its application for clinical use. Radiomics has emerged as a potential tool for identification of imaging biomarkers for cancer treatment, which could assist in clinical decision making and personalized medicine for patients with cancer.

## Figures and Tables

**Table 1:** Summary of radiomic literature on predicting response to neoadjuvant chemoradiotherapy in patients with rectal cancer.

Time	First author	Study design	No. of patients (training + testing)	No. and type of radiomic features	Feature selection model	Image modality	Segmentation method
2021 Mar 8 [25]	Delli Pizzi	Retrospective, single center study	72	1688 (first-order statistics, shape descriptors, GLCM, GLRLM, NGTDM, GLDM, and GLSZM)	Partial least square (PLS) regression	T2-W	Manual segmentation

2019 Mar [25]	Cui	Retrospective, single center study	131 + 55	1188 (first-order, size and shape, and texture)	LASSO. Logistic nomogram	T2-W Ct1-W ADC	Manual segmentation

2018 Jun [26]	Horvat	Retrospective, single center study	114	34 (texture)	RF, Wilcoxon rank-sum test, Benjamini–Hochberg method, and McNemar test	T2-W T2-W + DWI	Manual segmentation

2019 Apr [27]	Giannini	Retrospective single center study	52	30 (first-order texture, second-order texture)	Mann–Whitney test	T2-W, ADC, PET	Semiautomatic segmentation

2020 Apr [20]	Fu	Retrospective, single center study	43	105 (shape-based texture first-order) + 1472 (DL-based)	LASSO	DWI	Manual segmentation

2019 Mar [12]	Tang	Retrospective, single center study	152 + 70	563 (statistical, voxel-intensity computational, and wavelet)	DID regression analysis logistic regression	DWI	Manual segmentation

2020 Jan [28]	Bulens	prospective, single center study	70 + 55	2131 (intensity, shape and size, texture, and wavelet Gabor filters)	PCA and LASSO	T2-W	Manual segmentation

2020 May 21 [30]	Li	Retrospective, single center study	70 + 48	396 (42 first-order histogram features, 334 second-order texture features, 9 morphological features, and 11 gray-level zone size matrix features)	MRMR lasso	CT and MRI (DCE-T1, HR-T2WI, and ADC)	Manual segmentation

2019 Jun 26 [31]	Yi	Retrospective, single center study	94 + 40	340 (a gray-level histogram, gradient, run-length matrix, co-occurrence matrix, autoregressive model, and wavelet transform analysis according to the software settings.)	LASSO, RF, and SVM	T1-W, T2-W	Manual segmentation

2020 Jun [29]	Yuan	Retrospective, single center study	60 + 31	929 (Wavelet, texture)	Logistic, RF, and SVM	CT	Manual segmentation

2020 Nov [30]	Petkovska	Retrospective, single center study	66 + 36	108 (shape, texture)	Wilcoxon test, LASSO, and SVM	T2-W	Manual segmentation

2020 dec [31]	Karahan Şen	Retrospective single center study	88 + 22	44(texture, volumetric)	Mann–Whitney U test	18F-FDG PET/CT	Manual segmentation

2020 Aug [32]	Park	Retrospective single center study	78	First-order texture	Chi-square test, independent *t*-test, logistic regression, and cox regression	T2-W MRI	Manual segmentation

2020 Nov [33]	Martin-Gonzalez	Retrospective, single center study	37	33 (first-order, texture)	Stepwise multivariate binary logistic regression. Spearman nonparametric correlation test	18F-FDG PET	Manual segmentation

2021 Mar 25 [21]	Jin	Retrospective and prospective, multicenter study	321 + 301	Deep learning	Deep neural network architecture (3D RP-net).	T1\T2\DWI	Deep learning-based segmentation

2020 Aug [34]	Alvarez-Jimenez [21]	Retrospective, multicenter study	94(52 + 42)	389 (shape texture)	MRMR, RF, significance testing, and correlation testing	T2-W	Manual segmentation

2018 Feb [35]	Meng	Retrospective, single center study	59	First order and histogram texture metrics	LASSO	T2-W	Manual segmentation

2021 May [22]	Leng	prospective, single center study	32	Deep learning	CNN	PA PAM	Deep learning-based segmentation

2021 Feb [36]	Li	Retrospective, single center study	114	2818 (first-order shape, texture, and high-order)	ICC, the variance threshold algorithm, LASSO, and PCA	T2-W	Manual segmentation

2021 Mar [37]	Delli Pizz	Retrospective, single center study	72	10128 (first-order shape texture)	ICC, PLS	T2-W	Manual segmentation

2021 Jul [38]	Shayesteh	Retrospective, single center study	36 + 17	96 (intensity, morphological, and second- and high-order texture)	Max-relevance-min-redundancy (MRMR)	T2-W	Manual segmentation

2021 Aug [9]	Pang	Retrospective, multicenter study	196 + (46 + 34)	474 (intensity texture shape)	Harrell's concordance index, LASSO, and the univariate cox analysis	T2-W	Manual and deep learning-based segmentation

2021 Apr [39]	Liu	Retrospective, multicenter study	82 + 107	6192 (texture)	MRMR, RF	HR-T2WI and T1+C	Manual segmentation

2019 May 3 [40]	Shi	Prospective, single center study	51	96 (textural features and histogram-based features)	3-Layer perceptron artificial neural network (ANN)	T2-W, DWI, and DCEMRI	Manual segmentation

2019 Aug [41]	Shayesteh	Retrospective, single center study		80 (intensity, shape- and texture-based features, intensity direct, GLRLM, COM, and neighbor intensity difference)	Subset analysis (CF SUB E), correlation attribute evaluation (CO AT EV), gain ratio attribute evaluation (GA FA AT), one R attribute evaluation (One R AT) relief F attribute evaluation (RE F AT), and symmetrical uncertainty attribute evaluation (SYM AT)	T1-2, T2-W	Manual segmentation

Sep-oct 2020 [42]	Hyuck Jeon	Retrospective, single center study	90 + 45	65 (volume, first-order texture)	Intraclass correlation coefficient values	T2-W	Manual segmentation

2016 Nov [43]	Nie	Retrospective, single center study	103	103 (texture, histogram-based features, and shape)	ANN	T1/T2, DWI, and (DCE) MRI	Manual segmentation

2020 May [44]	Chen	Retrospective, single center study	39	294 (shape, first-order, high-order texture, and Laplacian of Gaussian filter-based features)	SVM and Wilcoxon rank-sum test	HR-T2WI	Manual segmentation

2020 Nov [45]	Shaish	Retrospective, multicenter study	132	1595 (first-order, texture, and shape)	Logistic regression	T2-W	Manual segmentation

2019 Sep [46]	Ferrari	Retrospective, single center study	28 + 27	855 (first-order, second-order)	RF and 2-tail t-student test	T2-W	Manual segmentation

2018 Aug [23]	Bibault	Retrospective, multicenter study	49 + 46	1683 (texture, shape)	Chi-squared tests and Wilcoxon tests	MRI, PET-CT	Manual segmentation

2020 Jul [47]	Petresc	Retrospective, single center study	44 + 23	960 (first-order, second-order)	Inter-reader agreement evaluation univariate analysis, spearman correlation analysis, and LASSO	T2-W	Manual segmentation

2020 Mar [48]	Shen	Retrospective, single center study	169	68 (classical PET features, and texture)	RF	18 F [FDG]-PET/CT	Manual segmentation

2020 Mar [49]	van Griethuysen	Retrospective, multicenter study	86 + 47	2505 (shape, intensity, and texture)	ICC, MRMR, and logistic regression	T2-W, DWI, ADC	Manual segmentation and semiautomatic

2020 Oct [50]	Palmisano	Prospective single center study	43	Whole-lesion (three-dimensional [3D]) first-order texture	Spearman correlation coefficient and Kruskal– Wallis test, post-hoc analysis with Dunn's test	T2-W	Manual segmentation

2021 Nov [51]	Wan	Retrospective, single center study	116 + 49	1049 (shape, first-order, texture, and high-order)	Standardization scaling the maximum relevance minimum redundancy algorithm, LASSO	T2-W	Manual segmentation

2020 Oct [3]	Zhu	Retrospective, single center study	500 + 200	Deep learning	Deep convolutional neural network	T2-W, DWI	Manual segmentation and semiautomatic

2019 Jun [52]	Shayesteh	Prospective single center	53 + 45	80 (intensity, shape and texture feature)	Pearson's chi-square X2 algorithm	T2-W	Manual segmentation

2019 Feb [53]	Boldrini	Retrospective, single center study	16	318 (morphological, statistical, fractal, and textural)	Wilcoxon–Mann–Whitney (WMW) test	Hybrid magnetic resonance	Manual segmentation

2021 Apr [54]	Cusumano	Retrospective, multicenter study	43	2 delta features	—	T2-W, T1-W	—

2021 Jan [55]	Cui	Retrospective, single center study	131 + 53	1130 (first-order, shape, texture, and wavelet and Laplacian of Gaussian (LoG))	ICC, the Boruta algorithm	Multiparameter MRI	Manual segmentation

2019 Dec [56]	Li	Retrospective, single center study	87 + 44	161 (volume, intensity, and texture)	LASSO	T2-W	Manual segmentation

2021 May [57]	Wang	Retrospective, single center study	122 + 61	942 (shape and texture)	MRMR and LASSO	Multiparametric MRI	Manual segmentation

2018 Dec [58]	van Helden	Retrospective, single center study	99	10 (texture and shape)	—	18F-FDG PET	Semiautomatic segmentation

2021 Dec [59]	Lutsyk	Retrospective, single center study	140	850 (texture and wavelet)	RF	CT	Manual segmentation

2021 Jun [60]	Zhuang	Prospective, single center study	118 + 59	1218 (first-order, texture)	LASSO. Linear combination of estimated coefficients and radiomic values	CT, T2-W	Manual segmentation

2021 Apr [61]	Coppola	Retrospective, single center study	40	84 (first-order)	RF	T2-W	Manual segmentation

2021 Nov [62]	Chiloiro	Retrospective, single center study	144	232 (statistical, morphological, and textural)	Wilcoxon–Mann–Whitney test, Pearson's correlation method, and logistic regression	T2-W	Manual segmentation

2021 Nov [63]	Cheng	Retrospective, single center study	129 + 64	1967 (first-order, texture, shape)	ICC, Mann–Whitney U test, LASSO, and the logistic regression with Akaike information criterion (AIC)	T1-W, T2-W, and T2FS	Manual segmentation

2019 Oct [64]	Hamerla	Retrospective, single center study	169	1819 (shape, first-order, and texture)	RF, recursive feature elimination	Noncontrast CT	Manual segmentation

2018 apr [65]	Cusumano	Retrospective and prospective multicenter study	173 + 25	Fractal features. Morphological features, statistical features	Wilcoxon–Mann–Whitney and Pearson's *χ*^2^ test	T2-W	Manual segmentation

2021 May [66]	Zhang	Retrospective, single center study	134 + 55	30 (texture)	ICC, MRMR, univariable logistic regression, and LASSO	T1-W and T2-W	Manual segmentation

2020 Nov [67]	Antunes	Retrospective, multicenter study	60 + 44	746 (texture)	Correlation pruning + Wilcoxon, MRMR, binarized LASSO	T2-W	Manual segmentation

2021 Nov [24]	Khadidos	Retrospective, single center study	98	1045-1632 and deep learning	RFE and RF (conventional learning model)	T2-W	Manual segmentation

2017 Dec [68]	Liu	Retrospective, single center study	152 + 70	2252 (first-order, and texture, wavelet)	Univariate statistical tests (two-sample *t*-test), LASSO	T2-W	Manual segmentation

2019 Jun [69]	Zhou	Retrospective, single center study	318 + 107	2424 (first-order statistics, GLCM textural features, and Laplacian of Gaussian (LoG) filtration features)	Wilcoxon rank-sum test, Spearman correlation analysis, and least absolute shrinkage and selection operator regression	Multiparametric MRI	Manual segmentation

2018 Mar [70]	Lovinfosse	Retrospective, single center study	86	First-order, texture	—	18F-FDG PET/CT	Automatic segmentation

2019 Jun [14]	Vandendorpe	Retrospective, multi-center study	79 + 42	66 (texture)	Best model (ELASTIC-NET method)	Contrast-enhanced computed tomography (CT)	Manual segmentation

2021 Mar [17]	Liu	Retrospective, single center study	198 + 83	378 (histogram, form factor, GLCM, RLM, and GLSZM features)	MRMR and LASSO	CT, T2-W, DWI, and CE-TIWI	Manual segmentation

2020 Apr [16]	Zhang	Retrospective, single center study	65 + 29	1188 (first order, morphological, haralick, GLZSM, GLCM, and RLM)	Student's t-test, Mann–Whitney U test, LASSO, and Spearman's rank correlation	CT and MRI	Manual segmentation

2019 Oct [71]	Wang	Retrospective, single center study	411	271 (wavelet, fraction demission, GLRLM, GLCM, histogram, and shape)	Test-retest and contour-recontour and Spearman's correction coefficient	CT	Manual segmentation

2020 Oct [15]	Nakanishi	Retrospective, multicenter study	175 + 72	1038 (first-order and shape)	LASSO	Contrast-enhanced abdominal CT	Manual segmentation

2020 Sep [18]	Li	Retrospective, single center study	103 + 45	396 (first-order, second-order, and higher-order)	LASSO	CT	Manual segmentation

2021 Feb [72]	Yuan	Retrospective, single center study	66	929 (intensity, shape, GLCM, GLRLM, GLSZM, NGTDM, LoG, wavelet, Laws, and Fractal dimension (FD).	Logistic regression and Pearson test	CT PET	Manual segmentation

2020 Jul [73]	Zhang	Prospective, single center	290 + 93	Deep learning	Multipath convolutional neural network	Diffusion kurtosis MRI	Manual segmentation

2021 Aug [74]	Jang	Retrospective, single center study	353 + 113	Deep learning	CNN, long short-term memory network (LSTM) model	MRI	Automatic segmentation

ANN = artificial neural network; ADC = apparent diffusion coefficient; COM = co-occurrence matrix features; CT = computed tomography; DID = difference in difference; DWI = diffusion weighted imaging; GLCM = gray-level co-occurrence matrix; GLRLM = gray-level run-length matrix; GLDM = gray-level difference method; GLSZM = gray-level size zone matrix; ICA = intraclass correlation coefficients; LASSO = least absolute shrinkage and selection operator; MRI = magnetic resonance imaging; MRMR = minimum redundancy maximum correlation; NGTDM = gray-tone difference matrix; PCA = principal component analysis; PET = positron emission tomography; PLS = partial least squares; RF = random forest; SVM = support vector machine.

**Table 2:** Comparison between classical radiomic methods and deep learning-based radiomic methods.

Methods	Steps	Advantages	Limitations
Classic radiomics	Image acquisition and preprocessing, tumor segmentation, feature extraction, feature selection, modeling, and validation	More commonly used and easier to perform	Labor-intensive and time-consuming
			Large inter-reader variability, information redundancy, and overfitting
Deep learning-based radiomics	Training of neural network structure	Saving time and efforts, more specific to clinical outcomes and data	Low interpretability

## Data Availability

The data used to support the findings of this study are available from the corresponding author upon request.
